# Cish attenuates proximal TCR-signaling and CD8+ T cell immunity

**DOI:** 10.1186/2051-1426-2-S3-P32

**Published:** 2014-11-06

**Authors:** Douglas Palmer, Geoffery Guittard, Shashank Patel, Lawrence E Samelson, Nicholas P Restifo

**Affiliations:** 1 National Cancer Institute, Bethesda, MD, United States

## 

CD8^+ ^T cells are potent killers of infected cells and tumors, specifically recognizing their targets through the T cell receptor (TCR). While much has been revealed about the activation of TCR signaling, negative regulation of this pathway remains incompletely elucidated. We report that Cish plays an inhibitory role in immunity to infection and adoptive transfer of *Cish*-deficient CD8^+ ^T cells eliminated established cancer. *Cish *deletion resulted in augmented acute phospholipase Cg1 (PLCg1) activation, Ca^2+ ^flux, NFAT and NFkB activity, and cytokine release. Conversely, *Cish *reconstitution decreased Ca^2+ ^flux, T cell polyfunctionality and PLCg1 accumulation in microclusters. We found that Cish physically interacts with PLCg1, targeting it for degradation following TCR engagement. Our data reveals a central role for Cish as a negative regulator of proximal TCR signaling and CD8^+ ^T cell immunity and identifies a new targetable interaction for immune-based therapy of infectious disease and cancer.

